# The Goldilocks principle: Finding the balance between water volume and nutrients for ovipositing *Culex* mosquitoes (Diptera: Culicidae)

**DOI:** 10.1371/journal.pone.0277237

**Published:** 2022-11-10

**Authors:** Noah C. Weidig, Amber L. Miller, Allison T. Parker

**Affiliations:** Department of Biological Sciences, Northern Kentucky University, Highland Heights, Kentucky, United States of America; University of Nevada Reno, UNITED STATES

## Abstract

Females of container-breeding mosquito species use visual and chemical cues to determine suitable habitats to oviposit their eggs. Female *Culex* mosquitoes oviposit single egg rafts containing hundreds of eggs on the surface of water in container habitats. In this project, the effects of water volume and nutrient concentration were studied using three semi-controlled field assays to determine the role these parameters play on female *Culex* mosquito oviposition preference. The results of this study suggest female *Culex* prefer to oviposit in larger volumes of water and higher concentrations of nutrients separately, but chose intermediate conditions when presented with a combination of these two variables, which follows the Goldilocks principle. This choice may provide their offspring with optimal conditions for development by reducing intraspecific competition, thereby maximizing the biological fitness of the ovipositing *Culex* females.

## Introduction

The Goldilocks principle is a phenomenon in which organisms select the intermediate value when presented with choices along a gradient [1, 2]. This principle is named after the tale, *Goldilocks and the Three Bears*. In this fairytale, Goldilocks comes across a house in the woods and tries out each of the three family member’s chairs, beds, and porridge. Goldilocks repeatedly chooses the option that falls not at either extreme of the range, but directly in the middle (“just right”). The Goldilocks Principle has been observed widely across the animal kingdom, including in insects [1–4]. For example, Enjin et al. (2016) demonstrated that *Drosophila melanogaster* prefer moderate humidity when presented with a range of humidity levels suggesting that the humidity preference for this fly species is in the intermediate range between too wet and too dry [3]. While the Goldilocks Principle has been observed in a range of animals, including insects, it has not been documented in mosquitoes.

Females of container-breeding mosquito species oviposit in a wide range of container habitats from naturally occurring tree holes to man-made flowerpots and garbage cans [5–8]. The resulting mosquito larvae developing within these container habitats compete for space and nutritional resources. The density of larvae and intensity of competition for space and nutritional resources within the container can affect adult life history traits including survival to adulthood, adult body size, and longevity [9–13]. Thus, it is important for the ovipositing females to select the optimum conditions within the container habitat to ensure survival to adulthood for their resulting offspring.

Oviposition choice is influenced by several environmental factors such as the volume and depth of water in the container, the nutrient concentration of that water, and the presence or absence of other mosquito larvae [5, 14, 15] The concentration of nutrients within a container habitat strongly affects oviposition choice. High nutrient concentrations provide ample resources for larval growth and development while minimizing intraspecific and interspecific competition [16–19]. However, excess nutrients can lead to high levels of microbial activity, creating biofilms that cover the surface of the water and prevent larval respiration [20]. Too little nutrients can hinder larval development, increase intraspecific and interspecific competition for nutrients, and lead to starvation [17, 18]. The volume and depth of water within the container can also influence oviposition choice [21, 22]. Too much water can lead to water spillover, and therefore potential spillover of larvae, out of the container during rain events [23]. Too little water can increase competition for space and food resources or lead to evaporation [24, 25]. The volume of water can also impact the concentration of nutrients within the habitat. Larger volumes of water can disperse nutrients decreasing the nutrient concentration whereas smaller volumes of water can create higher concentrations of nutrients even if the total nutrient availability is equal in both volumes of water [18]. Female mosquitoes must consider the interaction between nutrient availability and water volume when deciding which environment provides optimal resources for larval development.

*Culex* mosquitoes are container-breeding mosquitoes that oviposit a single egg raft on the surface of the water each gonotrophic cycle [26]. Egg rafts generally contain between 100 and 300 eggs [27]. Since *Culex* females lay all their eggs in one container, selecting the best habitat for all of her offspring to develop is key to increasing her fitness. Female *Culex* can sense numerous visual and olfactory cues of the container and water within the container, including water volume and nutrient concentration [28, 29]. Previous research suggests that female *Culex* prefer to oviposit in larger containers with greater volumes of water [6, 30], but some studies show that this preference is negated if nutrient levels are too low [19]. Since *Culex* females take both water volume and nutrients into consideration when deciding where to lay their eggs, females could be selecting the habitat that is “just right” for both water volume and nutrient concentration thereby following the Goldilocks Principle.

In this study, we examine how the interaction of water volume and nutrient concentration in identical container habitats influence female *Culex* oviposition choice. We hypothesized that females would examine both water volume and nutrient concentration and select the optimum combination of the two following the Goldilocks principle. Understanding how this interaction impacts female *Culex* oviposition choice provides deeper insight into the ecology of these mosquitoes.

## Materials and methods

### Ethics statement

Field collection and handling of mosquito eggs were conducted under a permit granted by the Northern Kentucky University: Research and Education Field Station (REFS) and the Campbell County Conservation District. All handling of mosquito eggs was conducted in accordance with institutional ethical standards.

### Study design

**Fig 1:**
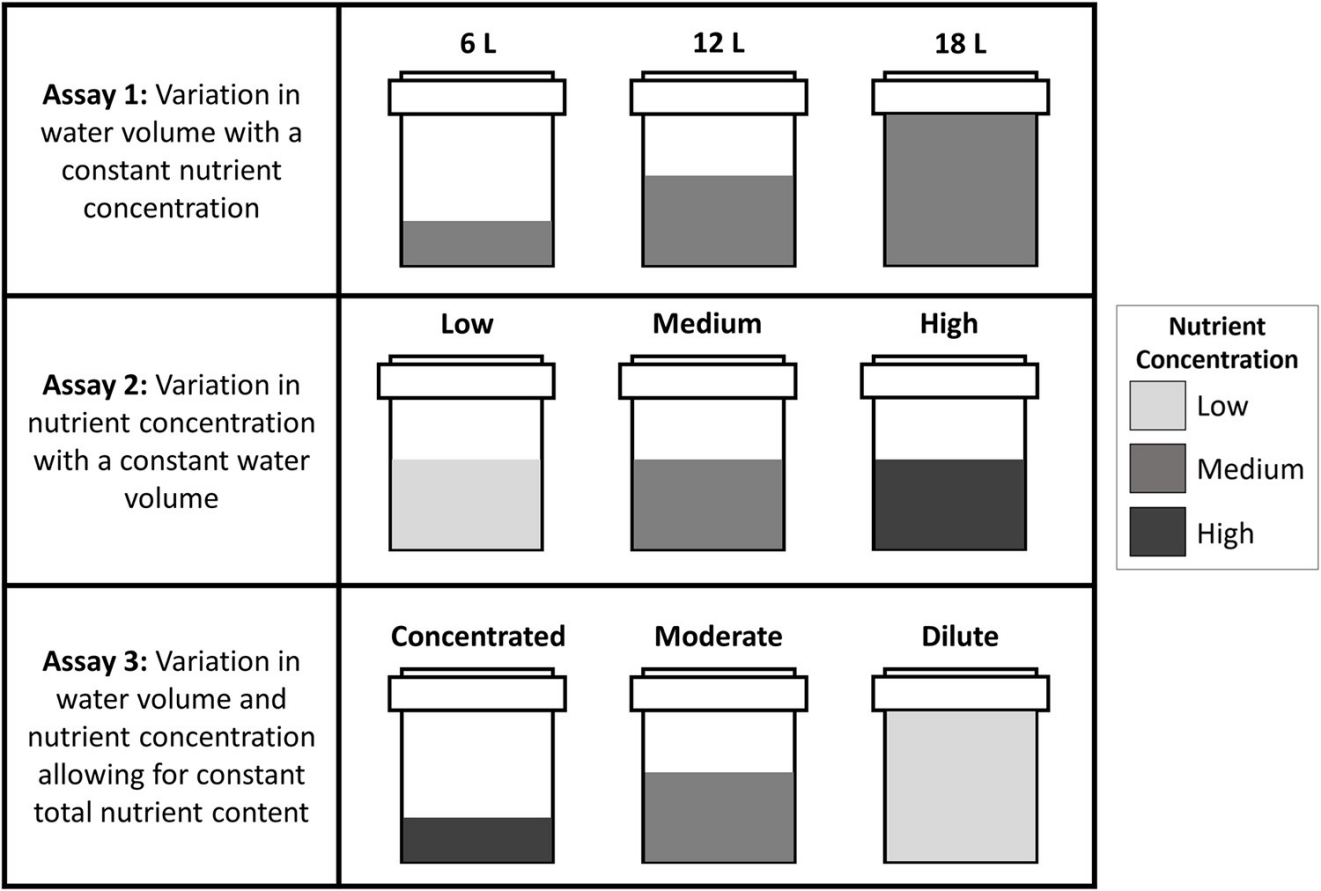
Visual representation of the three assays examining *Culex* female oviposition preferences for water volume and nutrient availability. Assay 1 examined variation in water volume (6L, 12L, 18L) using medium nutrient infusion in all containers. Assay 2 examined variation in total available nutrients (low, medium, high) across a constant water volume. Assay 3 examined variation in water volume and nutrient concentration (concentrated, moderate, dilute).

Three semi-controlled field assays were conducted at the Northern Kentucky University Research and Education Field Station (REFS), in Melbourne, Kentucky. At each of the fifteen field sites (each assay was conducted independently at five different sites), three container habitats (19-liter, white, cylindrical buckets) were evenly spaced in a triangle. To induce oviposition by *Culex* females, containers were filled with grass infusion to a predetermined water volume (6 liters (15 cm deep), 12 liters (25 cm deep), 18 liters (35 cm deep), depending on the assay). Three nutrient levels of Bermuda grass infusion were made using dried grass clippings wrapped in cheesecloth which were steeped in 185 liters of water in a 50-gallon rain barrel. Low nutrient infusion was made using 10 grams of grass clippings. Medium nutrient infusion was made using 20 grams of grass clippings. High nutrient infusion was made using 30 grams of grass clippings. Pilot studies were conducted to ensure adequate nutrition was available in each infusion level to support larval development. If evaporation had occurred, infusion was added daily to each bucket to maintain a constant volume. Once per week and after heavy rains, old infusion was removed and new grass infusion was added to each container. The three assay designs are as follows (Fig 1):

### Assay 1: Variation in water volume with a constant nutrient concentration

The first assay varied the water volume while keeping the nutrient concentration constant. At each of the five sites, one container held 6 liters of medium-nutrient grass infusion, one held 12 liters of medium-nutrient infusion, and one held 18 liters of medium-nutrient infusion.

### Assay 2: Variation in nutrient concentration with a constant water volume

The second assay varied the nutrient concentration while keeping the water volume constant. At each of the five sites, all three containers had the same water volume (12 liters) but varying nutrient concentrations (low, medium, and high).

### Assay 3: Variation in water volume and nutrient concentration allowing for constant total nutrient content

The third assay varied the water volume and nutrient concentration. This allowed for the total nutrient content available in the container habitat to remain constant across the treatments. At each of the five sites, one container held 6 liters of high-nutrient infusion, one held 12 liters of medium-nutrient infusion, and one held 18 liters of low-nutrient infusion (concentrated, moderate, dilute).

Every day for 12 weeks, the total number of egg rafts in each container was recorded, and a maximum of four egg rafts per container was collected for up to a total of 180 eggs rafts collected daily. All remaining egg rafts were removed from the container habitat daily. The collected egg rafts were allowed to hatch, and the emerged larvae were enumerated to species using taxonomic keys [31, 32].

### Statistical analysis

Generalized linear mixed-effects models (GLMERs) were used to analyze the fixed main effect of each assay (water volume in Assay 1, nutrient concentration in Assay 2, or total nutrient content in Assay 3) and the random effects of site by date on the total number of collected egg rafts. Overdispersion was assessed for each model [33]. No overdispersion was detected for Assay 1 (variation in water volume with a constant nutrient concentration) or for Assay 3 (variation in water volume and nutrient concentration allowing for constant total nutrient content). Since no overdispersion was found for Assay 1 or Assay 3, GLMERs using a Poisson regression were used. Overdispersion was detected for Assay 2 (variation in nutrient concentration with a constant water volume), so a quasiPoisson was used. Least square mean separation tests were used to detect significant pairwise differences between the three levels of each treatment for each assay. All analyses were carried out using an alpha level of 0.05 in R (version 3.5.1, R Core Team 2018, [34]) using RStudio (RStudio, Inc. Boston, MA 2016).

## Results

### Assay 1: Variation in water volume with a constant nutrient concentration

**Fig 2:**
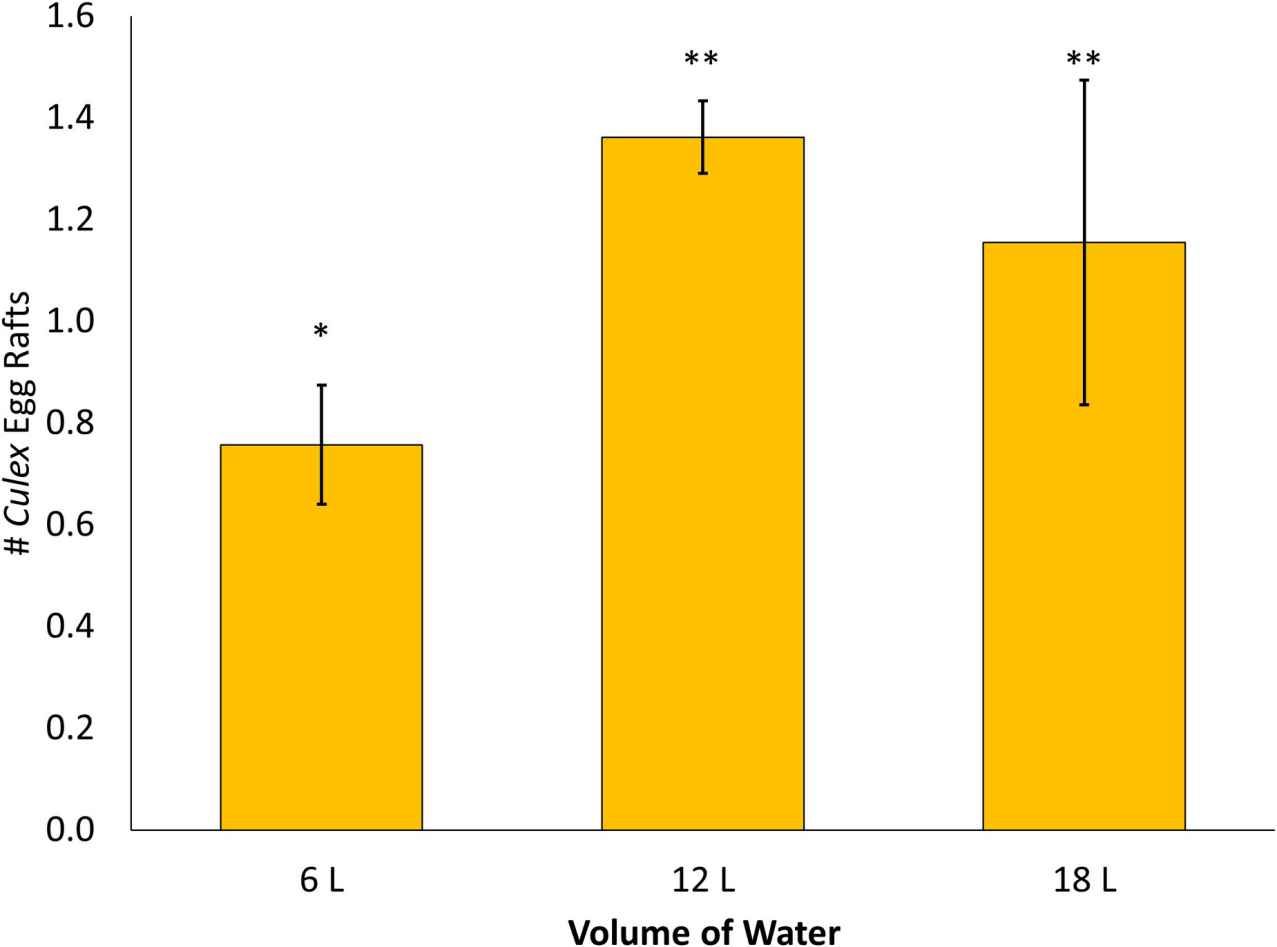
Number of egg rafts laid comparing three treatments with a constant nutrient content across multiple volumes of infusion. The three treatments in Assay 1 include 6 liters (6 liters of medium infusion), 12 liters (12 liters of medium infusion), and 18 liters (18 liters of medium infusion). Asterisks denote significant differences at P < 0.05.

The number of egg rafts laid was significantly influenced by the main effect of water volume (Table 2). Significantly fewer egg rafts were laid in the 6-liter treatment compared to the 12-liter and 18-liter volumes, and no significant difference was detected between the 12-liter and 18-liter volumes (Fig 2, Table 1).

**Table 1:** Poisson regression model constructed for number of egg rafts laid in Assay 1 comparing three treatments with a varied total nutrient content, fixed nutrient concentration across multiple volumes of infusion (6 liters: 6 liters of medium infusion, 12 liters: 12 liters of medium infusion, 18 liters: 18 liters of medium infusion).

	Estimate	Standard error	P-value
**Intercept**	-1.854	0.263	<0.001
**6 liters**	-0.449	0.073	<0.001
**12 liters**	0.167	0.062	<0.001

### Assay 2: Variation in nutrient concentration with a constant water volume

**Fig 3:**
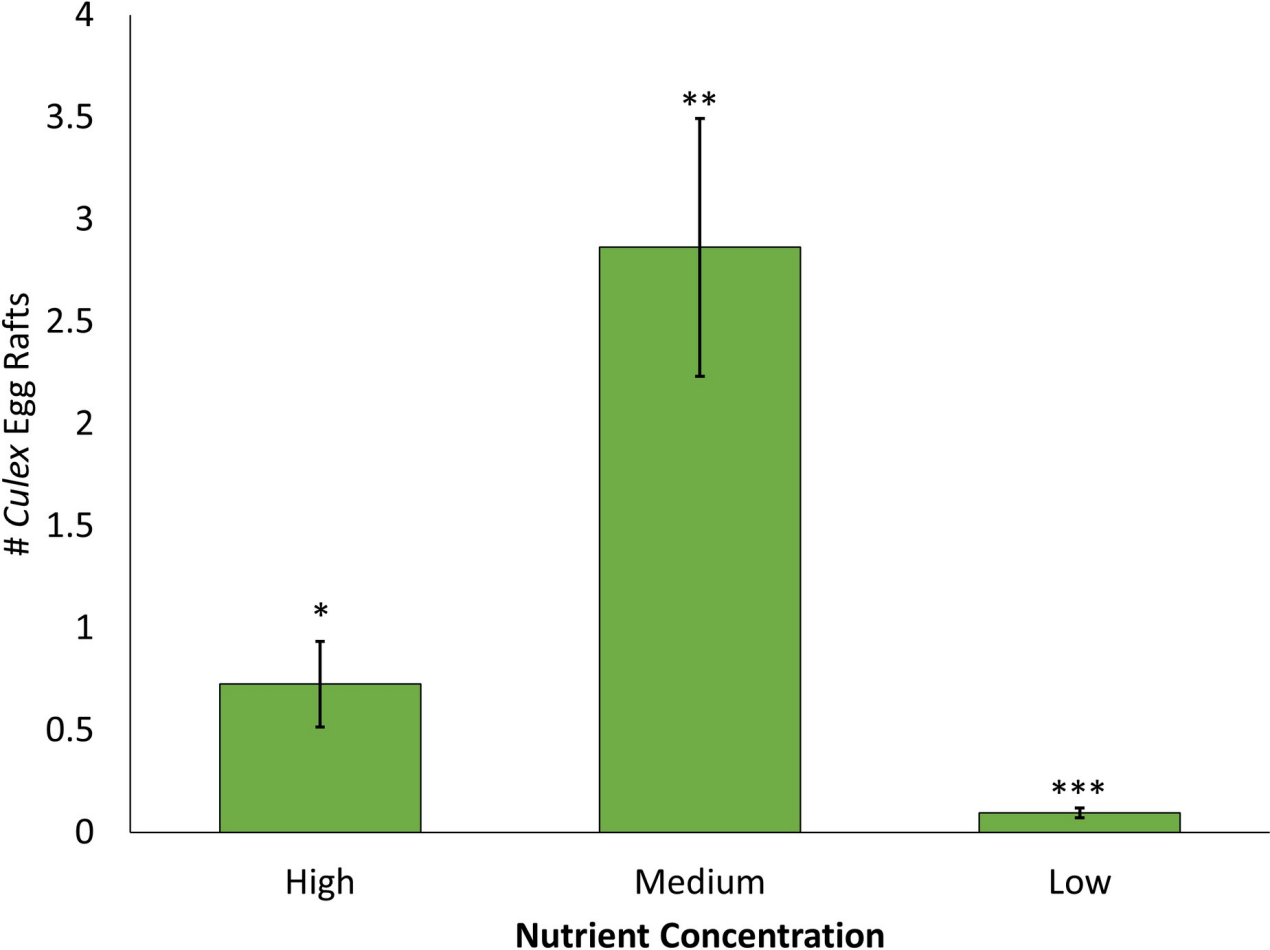
Number of egg rafts laid comparing three treatments with a variation in nutrient concentration with a fixed water volume. The three treatments in Assay 2 include Low (12 liters of low infusion), Medium (12 liters of medium infusion), and High (12 liters of high infusion). Asterisks denote significant differences at P < 0.05.

The number of egg rafts laid was significantly influenced by the main effect of nutrient concentration (Table 2). Significantly fewer egg rafts were laid in the low treatment compared to the medium and high treatments, and no significant difference was detected for the number of egg rafts laid between the medium and high treatments (Fig 3, Table 2).

**Table 2:** QuasiPoisson regression model constructed for number of egg rafts laid in Assay 2 comparing three treatments with a variation in nutrient concentration with a fixed water volume (Low: 12 liters of low infusion, Medium: 12 liters of medium infusion, High: 12 liters of high infusion).

	Estimate	Standard error	P-value
**Intercept**	-6.547	0.489	<0.001
**High**	2.031	0.168	<0.001
**Medium**	3.404	0.161	<0.001

### Assay 3: Variation in water volume and nutrient concentration allowing for constant total nutrient content

**Fig 4:**
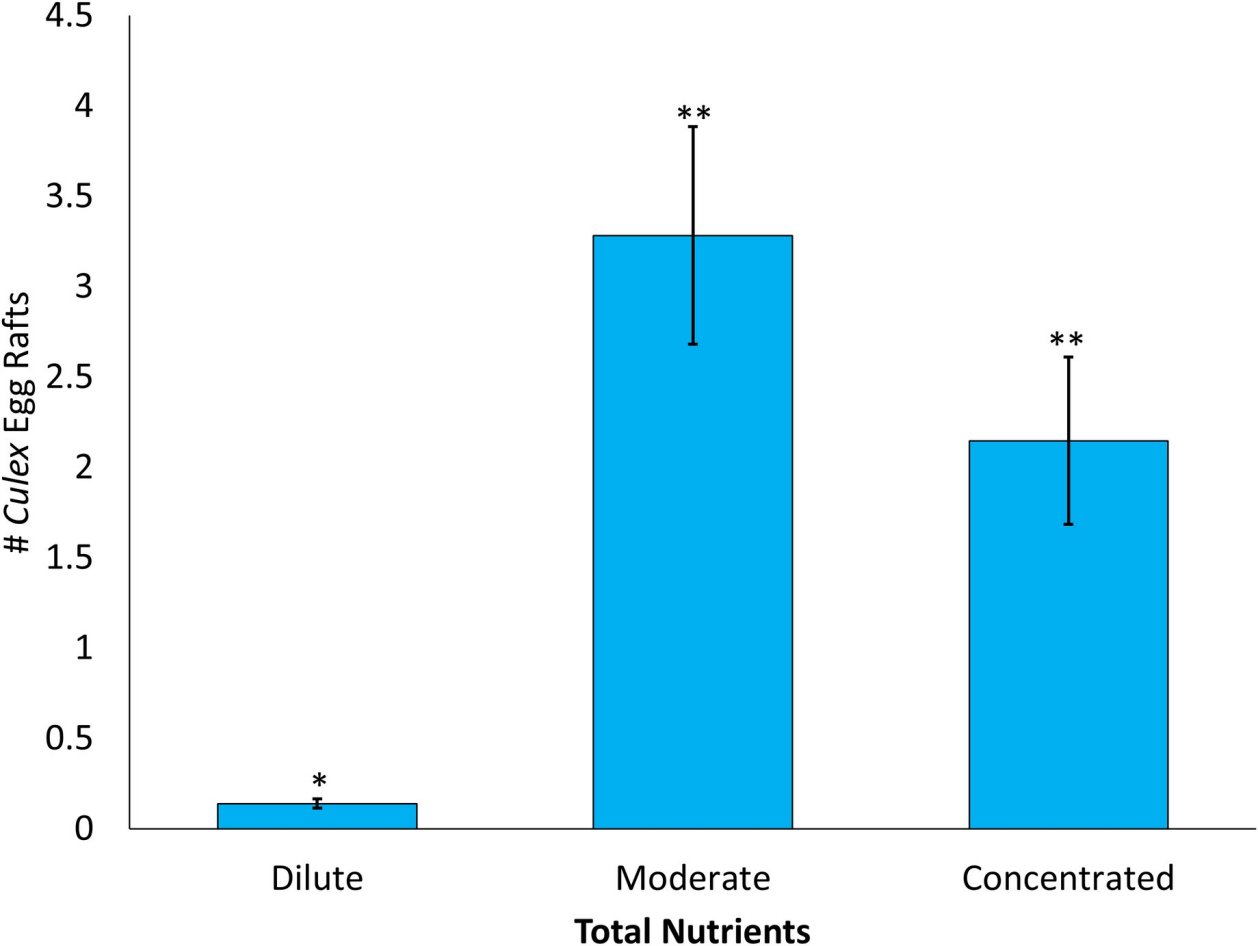
Number of egg rafts laid comparing three treatments with variation in water volume and nutrient concentration allowing for constant total nutrient content. The three treatments in Assay 3 include Concentrated (6 liters of high infusion), Moderate (12 liters of medium infusion), and Dilute (18 liters of low infusion). Asterisks denote significant differences at P < 0.05.

The number of egg rafts laid was significantly influenced by the main effect of total nutrient content (Table 3). The greatest number of egg rafts were laid in the moderate treatment, the second-most amount of egg rafts were laid in the concentrated treatment, and the lowest number of eggs rafts were laid in the dilute treatment (Fig 4, Table 3).

**Table 3:** Poisson regression model constructed for number of egg rafts laid in Assay 3 comparing three treatments with variation in water volume and nutrient concentration allowing for constant total nutrient content (concentrated: 6 liters of high infusion, moderate: 12 liters of medium infusion, dilute: 18 liters of low infusion).

	Estimate	Standard error	P-value
**Intercept**	-2.581	0.257	<0.001
**Dilute**	-2.496	0.260	<0.001
**Moderate**	0.227	0.183	0.215

## Discussion

The effects of nutrient concentration and water volume on oviposition choice have been studied separately in various experiments but studying the interaction between these two environmental parameters can be challenging. In this study, *Culex* females show that both water volume and nutrient concentration are being taken into account when deciding on the location where they oviposit their eggs. The results from this study suggest that female *Culex* are following the Goldilocks principle and looking for the container habitat that is the “just right” combination of water volume and nutrient concentration. Our results suggest that females showed a preference for a moderate volume of water and a medium nutrient concentration compared to high nutrients in a small volume of water or to a diluted concentration of nutrients in a large volume of water. This may be an evolutionary strategy to ensure sufficient nutrients for their offspring while preventing either the over dispersion of those nutrients in large container habitats or overcrowding and increased intraspecific competition in small volumes of water.

Previous research on the effects of water volume on oviposition choice suggests that females prefer to lay their egg rafts in larger volumes of water compared to smaller volumes. Parker et al. (2020) showed that *Culex* females prefer larger containers and thus larger volumes of water, and in the absence of the large containers, there was a significant decrease in the number of egg rafts laid by *Culex* females suggesting that the females choose to look for other, more suitable habitats [6]. However, the main limitation of this study was that there was no control for nutrients since the infusion used was the same across all container sizes. Thus, larger containers harbored more nutrients compared to smaller containers holding smaller volumes of water. In Assay 1 of this study, we found similar results where female *Culex* preferred to oviposit in containers with larger volumes of water, but in Assay 1, water volume was also conflated with nutrient availability with larger volumes of water having a greater amount of nutrients. Females may have been selecting containers with higher nutrient concentrations instead of larger water volumes. Additional assays in this study allowed for the examination of the interaction between these two parameters to better elucidate the driving factors in oviposition choice for *Culex* females.

The amount of nutrients and type of nutrients in the container habitat can also impact *Culex* oviposition preferences. Previous research shows that female *Culex* mosquitoes prefer to oviposit in containers harboring certain species of plant and animal detritus [35, 36] suggesting that females are using the chemical cues being emitted from the container habitat to determine habitat suitability for their offspring. In this project, Bermuda grass infusion was used in all treatments because it is a common plant that can enter into container habitats and grass infusion has been shown to be attractive to *Culex* mosquitoes [36, 37]. Using the same grass infusion across all containers and trials ensured that chemical cues from different plant species were not eliciting the observed oviposition choices. Oviposition rates have also been shown to be significantly higher in containers with higher nutrient concentrations of the same food resources [19, 38] suggesting that female *Culex* are inspecting both the strength and type of chemical cues being emitted from a container habitat. In Assay 2 of this project, water volume was held constant while nutrient concentration was varied. Our results suggest that females preferentially oviposit in containers with medium and high nutrient concentrations compared containers with low nutrient concentration when water volume is held constant. This shows that *Culex* females are taking nutrient concentration into consideration when deciding where to oviposit.

In addition to chemical cues, visual cues, such as water color and depth, also play an important role in oviposition choice for female mosquitoes. Water color may be an indication of the concentration of nutrients in the container habitat or of the water volume. *Culex* females have been shown to preferentially oviposit in containers with darker colored water suggesting that females may be using this visual cue as an initial indicator of nutrient concentration [39, 40]. Color of the container habitat has been shown to impact oviposition choice among female mosquitoes with females preferring darker-colored containers [41]. In this project, all the containers were white, which may have impacted the results of the study as *Culex* females may be using the visual cue of the water height in relationship to the exposed height of the white container as a way to determine suitable habitats for their offspring [42]. Two major limitations of this study was that the height of the water to the top of the container and water depth was not fixed but varied with water volume with smaller volumes of water being lower in the container habitat and having a smaller depth of water and larger volumes of water coming closer to the top of the container and having a greater depth of water. A future experiment could include controlling for water surface height to eliminate variation based on water depth. This would indicate whether the height of the water in relationship to the height of the container plays a role in oviposition choice. A different result may have been observed if each container habitat had the same distance from the surface of the water to the top of the container but with different water volumes. Since water depth was also confounded with water volume, another future experiment may examine how changes in water volume while keeping water depth constant (changes in pool size) can impact oviposition choice.

Since no parental care is provided for the developing *Culex* larvae, the ability of adult females to select the highest quality habitat for their offspring is essential for achieving high levels of biological fitness. The results of this study show that *Culex* females are indeed looking at the interaction between nutrients and water volume when deciding where to oviposit. Higher nutrient content allows for faster development and greater survivorship to adulthood [17, 43]. Females may also be selecting container habitats that buffer potential competition between their offspring and other larvae developing in the container habitat. In some cases, superior competitors such as *Aedes albopictus* lose their competitive advantage at higher nutrient levels or due to certain nutrients being available within the container habitat [44, 45] and larger volumes of water in a habitat might allow for higher nutrient concentrations since larger volumes of water typically occur in larger container habitats. Thus, female *Culex* may be selecting larger volumes of water while also having sufficient nutrients to allow for a reduction in the competitive advantage of superior larval competitor leading to the observation of the Goldilocks principle for ovipositing female *Culex* mosquitoes. Elucidating the role of oviposition choice in the distribution of mosquito species can aid in our understanding of overall mosquitoes species distribution.

## References

[1] Sankey DWE, Shepard ELC, Biro D, Portugal SJ. Speed consensus and the ‘Goldilocks principle’ in flocking birds (*Columba livia*). Anim Behav. 2019;157: 105–119. doi: 10.1016/j.anbehav.2019.09.001

[2] Heimpel GE, Asplen MK. A ‘Goldilocks’ hypothesis for dispersal of biological control agents. BioControl. 2011;56: 441–450. doi: 10.1007/s10526-011-9381-7

[3] Enjin A, Zaharieva EE, Frank DD, Mansourian S, Suh GSB, Gallio M, et al. Humidity sensing in *Drosophila*. Curr Biol. 2016;26: 1352–1358. doi: 10.1016/j.cub.2016.03.049 27161501 PMC5305172

[4] Somero GN. The Goldilocks Principle: A unifying perspective on biochemical adaptation to abiotic stressors in the sea. Annu Rev of Mar Sci. 2022;14: 1–23. doi: 10.1146/annurev-marine-022521-102228 34102065

[5] Leisnham PT, LaDeau SL, Saunders MEM, Villena OC. Condition-specific competitive effects of the invasive mosquito *Aedes albopictus* on the resident *Culex pipiens* among different urban container habitats may explain their coexistence in the field. Insects. 2021;12: 993. doi: 10.3390/insects12110993 34821793 PMC8621322

[6] Parker AT, McGill K, Allan BF. Container type affects mosquito (Diptera: Culicidae) oviposition choice. J Med Entomol. 2020;57: 1459–1467. doi: 10.1093/jme/tjaa045 32161973

[7] Low R, Boger R, Nelson P, Kimura M. GLOBE Mosquito Habitat Mapper citizen science data 2017–2020. GeoHealth. 2021;5: e2021GH000436. doi: 10.1029/2021GH000436 34712882 PMC8527845

[8] Bartlett-Healy K, Unlu I, Obenauer P, Hughes T, Healy S, Crepeau T, et al. Larval mosquito habitat utilization and community dynamics of *Aedes albopictus* and *Aedes japonicus* (Diptera: Culicidae). J Med Entomol. 2012;49: 813–824. doi: 10.1603/ME11031 22897041

[9] Walker M, Chandrasegaran K, Vinauger C, Robert MA, Childs LM. Modeling the effects of *Aedes aegypti’s* larval environment on adult body mass at emergence. PLOS Comput Biol. 2021;17: e1009102. doi: 10.1371/journal.pcbi.1009102 34807904 PMC8608295

[10] Bevins SN. Invasive mosquitoes, larval competition, and indirect effects on the vector competence of native mosquito species (Diptera: Culicidae). Biol Invasions. 2008;10: 1109–1117. doi: 10.1007/s10530-007-9188-8

[11] Juliano SA, Lounibos LP, O’Meara GF. A field test for competitive effects of *Aedes albopictus* on *A*. *aegypti* in South Florida: differences between sites of coexistence and exclusion? Oecologia. 2004;139: 583–593. doi: 10.1007/s00442-004-1532-4 15024640 PMC1906877

[12] Braks MAH, Honório NA, Lounibos LP, Lourenço-De-Oliveira R, Juliano SA. Interspecific competition between two invasive species of container mosquitoes, *Aedes aegypti* and *Aedes albopictus* (Diptera: Culicidae), in Brazil Ann Entomol Soc Am. 2004;97: 130–139. doi: 10.1603/0013-8746(2004)097[0130:ICBTIS]2.0.CO;2

[13] Reiskind MH, Lounibos LP. Effects of intraspecific larval competition on adult longevity in the mosquitoes *Aedes aegypti* and *Aedes albopictus*. Med Vet Entomol. 2009;23: 62–68. doi: 10.1111/j.1365-2915.2008.00782.x 19239615 PMC2651082

[14] Schoelitsz B, Mwingira V, Mboera LEG, Beijleveld H, Koenraadt CJM, Spitzen J, et al. Chemical mediation of oviposition by *Anopheles* mosquitoes: a push-pull system driven by volatiles associated with larval stages. J Chem Ecol. 2020;46: 397–409. doi: 10.1007/s10886-020-01175-5 32240482 PMC7205850

[15] Wachira SW, Ndung’u M, Njagi PGN, Hassanali A. Comparative responses of ovipositing *Anopheles gambiae* and *Culex quinquefasciatus* females to the presence of *Culex* egg rafts and larvae. Med Vet Entomol. 2010;24: 369–374. doi: 10.1111/j.1365-2915.2010.00913.x 21058965

[16] van Schoor T, Kelly ET, Tam N, Attardo GM. Impacts of dietary nutritional composition on larval development and adult body composition in the Yellow Fever mosquito (*Aedes aegypti*). Insects. 2020;11: 535. doi: 10.3390/insects11080535 32824225 PMC7469193

[17] Alto BW, Muturi EJ, Lampman RL. Effects of nutrition and density in *Culex pipiens*. Med Vet Entomol. 2012;26: 396–406. doi: 10.1111/j.1365-2915.2012.01010.x 22390256

[18] Parker AT, Gardner AM, Perez M, Allan BF, Muturi EJ. Container size alters the outcome of interspecific competition between *Aedes aegypti* (Diptera: Culicidae) and *Aedes albopictus*. J Med Entomol. 2019;56: 708–715. 10.1093/jme/tjy215. 30566608

[19] Reiskind MH, Walton ET, Wilson ML. Nutrient-dependent reduced growth and survival of larval *Culex restuans* (Diptera: Culicidae): Laboratory and field experiments in Michigan. J Med Entomol. 2004;41: 650–656. doi: 10.1603/0022-2585-41.4.650 15311456

[20] Hunt CM, Collins CM, Benedict MQ. Measuring and reducing biofilm in mosquito rearing containers. Parasite Vector. 2020;13: 439. doi: 10.1186/s13071-020-04315-8 32878628 PMC7466484

[21] Wynn G, Paradise CJ. Effects of microcosm scaling and food resources on growth and survival of larval *Culex pipiens*. BMC Ecology. 2001;1: 3. doi: 10.1186/1472-6785-1-3 11527507 PMC45585

[22] Yee DA, Abuzeineh AA, Ezeakacha NF, Schelble SS, Glasgow WC, Flanagan SD, et al. Mosquito larvae in tires from Mississippi, United States: The efficacy of abiotic and biotic parameters in predicting spatial and temporal patterns of mosquito populations and communities. J Med Entomol. 2015;52: 394–407. doi: 10.1093/jme/tjv028 26334813 PMC4581486

[23] Koenraadt CJM, Harrington LC. Flushing effect of rain on container-inhabiting mosquitoes *Aedes aegypti* and *Culex pipiens* (Diptera: Culicidae). J Med Entomol. 2008;45: 28–35. doi: 10.1093/jmedent/45.1.28 18283939

[24] Bartlett-Healy K, Healy SP, Hamilton GC. A model to predict evaporation rates in habitats used by container-dwelling mosquitoes. Inl Med Entom. 2011;48: 712–716. doi: 10.1603/ME10168 21661337

[25] Sota T, Mogi M, Hayamizu E. Habitat stability and the larval mosquito community in treeholes and other containers on a temperate island. Res Popul Ecol. 1994;36: 93–104. doi: 10.1007/BF02515090

[26] Madder DJ, Surgeoner GA, Helson BV. Number of generations, egg production, and developmental time of *Culex pipiens* and *Culex restuans* (Diptera: Culicidae) in southern Ontario. J Med Entomol. 1983;20: 275–287. doi: 10.1093/jmedent/20.3.275 6876091

[27] Vinogradova EB. *Culex Pipiens Pipiens* mosquitoes: Taxonomy, distribution, ecology, physiology, genetics, applied importance and control. Pensoft Publishers; 2000.

[28] Dhanalakshmi P, Sankaraiyah K, Kavipriya J, Vijayalakshmi MNV. Distribution of sensory structures on antennae of female *Culex* mosquito: A SEM study. J Entomol Zool Stud. 2018;6: 1900–1903.

[29] Ortiz-Perea N, Gander R, Abbey O, Callaghan A. The effect of pond dyes on oviposition and survival in wild UK *Culex* mosquitoes. Lorenzo MG, editor. PLoS ONE. 2018;13: e0193847. doi: 10.1371/journal.pone.0193847 29590133 PMC5873999

[30] Daniels S, Ezeakacha NF, Yee DA. Interspecific interactions between adult *Aedes albopictus* and *Culex quinquefasciatus* (Diptera: Culicidae). J Med Entomol. 2016;53: 466–469. doi: 10.1093/jme/tjv188 26628685 PMC5853674

[31] Ross HH, Horsffall WR. A synopsis of the mosquitoes of Illinois (Diptera, Culicidae). Biological Notes. 1965; 50.

[32] Andreadis TG. Identification guide to the mosquitoes of Connecticut. Connecticut Agricultural Experiment Station. 2005.

[33] Korner-Nievergelt F, Roth T, von Felten S, Guelat J, Almasi B, Korner-Nievergelt P. blmeco: Data files and functions accompanying the book “Bayesian data analysis in ecology using R, BUGS and Stan.” 2019. Available: https://CRAN.R-project.org/package=blmeco.

[34] R Core Team. R: A language and environment for statistical computing. R Foundation for Statistical Computing. 2018.

[35] Gardner AM, Allan BF, Frisbie LA, Muturi EJ. Asymmetric effects of native and exotic invasive shrubs on ecology of the West Nile virus vector *Culex pipiens* (Diptera: Culicidae). Parasite Vector. 2015;8: 329. doi: 10.1186/s13071-015-0941-z 26076589 PMC4469247

[36] Jackson BT, Paulson SL, Youngman RR, Scheffel SL, Hawkins B. Oviposition preferences of *Culex restuans* and *Culex pipiens* (Diptera: Culicidae) for selected infusions in oviposition traps and gravid traps. J Am Mosq Control Assoc. 2005;21: 360–365. doi: 10.2987/8756-971X(2006)21[360:OPOCRA]2.0.CO;2 16506560

[37] Millar JG, Chaney JD, Mulla MS. Identification of oviposition attractants for *Culex quinquefasciatus* from fermented Bermuda grass infusions. J Am Mosq Control Assoc. 1992;8: 11–17. 1583482

[38] Reiskind MH, Wilson ML. *Culex restuans* (Diptera: Culicidae) oviposition behavior determined by larval habitat quality and quantity insSoutheastern Michigan. J Med Entomol. 2004;41: 179–186. doi: 10.1603/0022-2585-41.2.179 15061276

[39] Li J, Deng T, Li H, Chen L, Mo J. Effects of water color and chemical compounds on the oviposition behavior of gravid *Culex pipiens pallens* females under laboratory conditions. J Ag Urban Entomol. 2009;26: 23–30. doi: 10.3954/1523-5475-26.1.23

[40] Beehler JW, Millar JG, Mulla MS. Synergism between chemical attractants and visual cues influencing oviposition of the mosquito, *Culex quinquefasciatus* (Diptera: Culicidae). J Chem Ecol. 1993;19: 635–644. doi: 10.1007/BF00984998 24249007

[41] Torrisi GJ, Hoback WW. Color and container size affect mosquito (*Aedes triseriatus*) oviposition. Northeastern Naturalist. 2013;20: 363–371. doi: 10.1656/045.020.0211

[42] Shin D, O’Meara GF, Civana A. Size of openings in water-holding containers: Impact on oviposition by *Culex* Mosquitoes. Insects. 2019;10: 257. doi: 10.3390/insects10090257 31438538 PMC6780729

[43] Noori N, Lockaby BG, Kalin L. Larval development of *Culex quinquefasciatus* in water with low to moderate. J Vector Ecol. 2015;40: 208–220. doi: 10.1111/jvec.12156 26611953

[44] Costanzo KS, Muturi EJ, Lampman RL, Alto BW. The effects of resource type and ratio on competition with *Aedes albopictus* and *Culex pipiens* (Diptera: Culicidae). J Med Entomol. 2011;48: 29–38. doi: 10.1603/ME10085 21337945

[45] Müller R, Knautz T, Vollroth S, Berger R, Kreß A, Reuss F, et al. Larval superiority of *Culex pipiens* to *Aedes albopictus* in a replacement series experiment: prospects for coexistence in Germany. Parasite Vector. 2018;11: 80. doi: 10.1186/s13071-018-2665-3 29394910 PMC5797359

